# A General Synthesis of Bis-indolylpiperazine-2,5-diones

**DOI:** 10.3390/molecules171214841

**Published:** 2012-12-13

**Authors:** Stephen Crooke, Christine Whitlock

**Affiliations:** 1 Department of Chemistry and Biochemistry, Georgia Institute of Technology, 901 Atlantic Drive, Atlanta, GA 30332, USA; Email: scrooke@gatech.edu; 2 Department of Chemistry, Georgia Southern University, P.O. Box 8064, Statesboro, GA 30460, USA

**Keywords:** indole, bromination, dragmacidin derivatives

## Abstract

The one-pot synthesis of three dragmacidin derivatives is reported. Sarcosine anhydride (**4**) is brominated and immediately reacted with the corresponding indole to produce the products, namely 3,6-bis(5′-methoxy-3′-indolyl)-1,4-dimethylpiperazine-2,5-dione (**1**), 3,6-bis(7′-methyl-3′-indolyl)-1,4-dimethylpiperazine-2,5-dione (**2**) and 3,6-bis-(6′-chloro-3′-indolyl)-1,4-dimethylpiperazine-2,5-dione (**3**), which are characterized by ^1^H-NMR.

## 1. Introduction

During the past twenty years, a number of natural products containing the indole moiety that display a significant amount of biological activity have been isolated from marine organisms [1,2,3,4]. Among these is a rapidly expanding class of bisindole alkaloid compounds isolated from marine sponges sp. *Dragmacidon*, *Spongosorites*, and *Hexadella*. Classified as dragmacidins [5,6], these compounds contain substituted indole residues joined by a piperazine linker at the 3-and 6-positions [7]. They have received considerable amounts of attention in the scientific community due to their applications as antiviral, antibacterial, anti-inflammatory, and cytotoxic agents [8,9,10,11,12,13,14,15,16,17,18,19].

Evidence supporting the need for further investigation into the activity of the dragmacidin compounds has arisen from multiple arenas. In relation to the cytotoxic activity of the dragmacidin class, it has been shown that serine-threonine protein phosphatases were significantly inhibited by dragmacidin D [20]. Dragmacidin D has also been shown to act as a potent inhibitor *in vivo* of resiniferitoxin-induced inflammation in murine ear tissue, as well as a selective inhibitor of neural nitric oxide synthase [21,22]. Dragmacidin has been reported to inhibit the *in vitro* growth of P388 (murine leukemia), A-549 (human lung), HCT-8 (human colon), and MDAMB (human mammary) cancer cell lines [8]. The broad range of abilities displayed by the dragmacidin compounds could prove useful in the development of cancer therapeutics and anti-inflammatory drugs, as well as prospective treatments for Alzheimer’s, Parkinson’s, and Huntington’s diseases [23] and depression and anxiety [24]. It is evident from the current studies that an effective method for synthetically producing dragmacidin derivatives would be beneficial.

Current synthetic methods center on the sequential implementation of palladium(0)-catalyzed Suzuki and Stille cross-coupling reactions [25,26]. A transamidation-cyclization has also been proposed as a synthetic method for the hamacanthin alkaloids [6]. These methods have proved to be moderately successful, but are impractical to implement on a large scale due to the stringent conditions required during multiple steps of the reactions. A short step, one-pot synthetic method was developed in our lab in order to prepare a dragmacidin intermediate [19]. This method was utilized to prepare novel dragmacidin precursors.

## 2. Results and Discussion

**Scheme 1:**
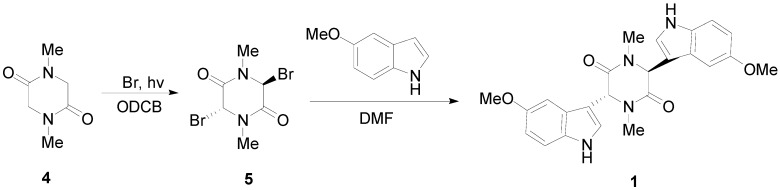
Synthesis of 3,6-bis(5′-methoxy-3′-indolyl)-1,4-dimethylpiperazine-2,5-dione (**1**).

**Scheme 2:**
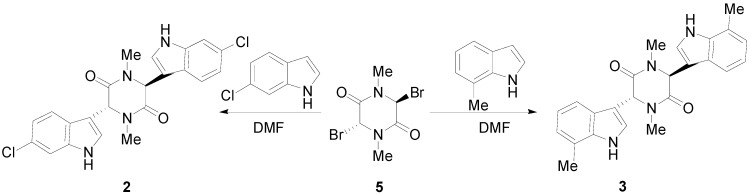
Syntheses of 3,6-bis(6′-chloro-3′-indolyl)-1,4-dimethylpiperazine-2,5-dione (**2**) and 3,6-bis(7′-methyl-3′-indolyl)-1,4-dimethylpiperazine-2,5-dione (**3**).

We now describe the synthesis of three novel bisindolylpiperazine-2,5-diones using a previously developed method [18]. The synthesis of 3,6-bis(5′-methoxy-3′-indolyl)-1,4-dimethylpiperazine-2,5-dione (**1**) is shown in Scheme 1. Bromine is directly added to sarcisine anhydride (**4**) with heat and the illumination of a sun lamp. After one hour, the solution is cooled to provide the dibrominated product as an unstable precipitate. This precipitate is then reacted with the corresponding indole in DMF to produce **1**, **2**, or **3**. Using a similar approach, 3,6-bis(6′-chloro-3′-indolyl)-1,4-dimethylpiperazine-2,5-dione (**2**), and 3,6-bis(7′-methyl-3′-indolyl)-1,4-dimethylpiperazine-2,5-dione (**3**) were prepared (Scheme 2). In conclusion, an important precursor to a dragmacidin derivative has been prepared by efficient means.

## 3. Experimental

*General*

Melting points are uncorrected. ^1^H-NMR spectra were obtained using a Bruker 250 MHz multi-nuclear spectrometer. Sarcosine anhydride (99.5%), 5-methoxyindole (99%), 6-chloroindole (99%), 7-methylindole (99%) were obtained from Acros Organics.

*3,6-Bis(5′-methoxy-3′-indolyl)-1,4-dimethylpiperazine-2,5-dione* (**1**). *Step 1. Preparation of 3,6-dibromo-1,4-dimethylpiperazine-2,5-dione* (**5**). Br_2_ (3.0 mL, 58.1 mmol) in *o*-dichlorobenzene (6 mL), was added dropwise under illumination of a sun lamp to a solution of sarcosine anhydride (**4**, 3.75 g, 26.4 mmol) in *o*-dichlorobenzene (15 mL), at 150 °C. The solution was heated for 1 h and then cooled to rt. A stream of N_2_ was bubbled through the reaction mixture for 10 min, and the solution was diluted with hexanes. The resulting beige crystals of **5** (an unstable precipitate) were filtered and rinsed with hexanes (7.06 g, 89.1%): mp 128–132 °C (lit. [27] 139–143 °C. ^1^H-NMR (CDCl_3_): 3.10 (s, 3H), 6.01 (s, 1H).

*Step 2.* Compound **5** (1.88 g, 6.27 mmol) was slowly added to a solution of 5-methoxyindole (1.00 g, 6.79 mmol) in DMF (15 mL), while the reaction temperature was maintained at room temperature with a water bath. The reaction mixture was stirred for 24 h, concentrated and diluted with methanol. The resulting solid was filtered to yield the product **1** as a white crystalline solid (0.33 g; 22.4%): mp > 250 °C. ^1^H-NMR (d_6_-DMSO): 2.66 (s, 3H), 3.27 (s, 3H), 5.58 (s, 1H), 6.82 (dd, 1H, *J* = 2.4, 8.8), 6.97 (d, 1H, *J* = 2.6), 7.33 (d, 1H, *J* = 28.8), 7.46 (d, 1H, *J* = 2.6), 11.09 (bs, 1H). 

*3,6-Bis(6′-chloro-3′-indolyl)-1,4-dimethylpiperazine-2,5-dione* (**2**). To a solution of 6-chlorolindole (1.00 g, 6.60 mmol) in DMF (15 mL) was slowly added **5**, while the reaction temperature was maintained at room temperature with a water bath. The reaction mixture was stirred for 24 h, concentrated and diluted with methanol. The resulting solid was filtered to yield the product **7** as a white crystalline solid (0.28 g; 19.4%): mp > 250 °C. ^1^H-NMR (d_6_-DMSO): 2.57 (s, 3H), 5.66 (s, 1H), 7.09 (d, 1H, *J* = 8.9), 7.22 (s, 1H), 7.51 (d, 1H, *J* = 6.7), 7.53 (d, 1H, *J* = 6.1), 11.23 (bs, 1H). 

*3,6-Bis(7′-methyl-3′-indolyl)-1,4-dimethylpiperazine-2,5-dione* (**3**). To a solution of 7-methylindole (1.00 g, 7.62 mmol) in DMF (15 mL) was slowly added **5**, while the reaction temperature was maintained at room temperature with a water bath. The reaction mixture was stirred for 24 h, concentrated and diluted with methanol. The resulting solid was filtered to yield the product **6** as a white crystalline solid (0.35 g; 23.1%): mp > 250 °C. ^1^H-NMR (d_6_-DMSO): ^1^H NMR (d_6_-DMSO): 2.49 (s, 3H), 3.36 (s, 3H), 5.63 (s, 1H), 6.95 (d, 2H, *J* = 2.9), 7.32 (d, 1H, *J* = 3.1), 7.49 (s, 1H), 11.20 (s, 1H). 

## 4. Conclusions

We have developed a reproducible method for preparing bis-indolylpiperazine-2,5-diones. These symmetrical compounds can serve as precursors in the development of biologically active alkaloids related to the dragmacidon, spongosorites, and hexadella series. 
